# Notes and News

**Published:** 1888-07-14

**Authors:** 


### NOTES AND NEWS.

Collected and Communicated.

Mr. R. Gofton Salmond, secretary of the British Home for Incurables, 380, Clapham Road, S.W., and 73, Cheapside, E.C., and originator of the Anglo-Danish Exhibition for the benefit of that charity, having completely broken down in health, has been ordered away for a three months' entire rest. Mr. John H. A. Langton has been appointed by the Board Acting Secretary for that period.

The report of the annual meeting of the Louth Hospital, which has been sent to us, discloses a satisfactory balance of £33 14*s.* 11*d.* in favour of the hospital at the close of the financial year. The Mayor (Mr. Alderman Barton) presided, and there were present, among others, Mr. W. Allinson (hon. treasurer), Mr. H. F. V. Falkner (hon. secretary), Rev. E. L. Gardener, Rev. Dr. Slater, Messrs. W. H. Smyth, C. W. Bell, W. Potter, Dr. Domenichetti, and others. It is proposed to build, in the near future, separate wards for infectious cases.

Ten thousand epileptic seizures experienced by one patient, was the remarkable statement made by the Right Rev. Bishop Perry when presiding at the annual meeting of the Governors and supporters of the Hospital for Epilepsy and Paralysis. After treatment the number gradually diminished. There was then a relapse, but ultimately the disease disappeared altogether. The story reads almost like a quack advertisement; but happily it is as true as it is extraordinary. The hospital, we regret to say, is in financial straits, and its much-needed development is hindered on this account.

On Thursday afternoon the National Hospital for the Paralyzed and Epileptic, Queen Square, Bloomsbury, following the example of many of its sister institutions in the metropolis, held a fête in commemoration of the Silver Wedding of the Prince and Princess of Wales. The celebration consisted of an entertainment, concert, flower show, a general floral decoration of the whole hospital, and a floral competition between the wards. The Princess Louise, who had accepted the invitation of the management, was received at the door by a deputation of the board of management and medical staff, among whom were the Lord Chancellor, the Hon. Dudley Ryder, Mr. F. Semon, and the Rev. J. H. Moran (chaplain). She was immediately conducted through the different wards, where evidence of the handiwork of the nurses was abundantly apparent in the beautiful floral wreaths and sprays which met the eye at every turn. The patients in these wards, who are sufferers from the various kinds of brain and nervous diseases, evidently appreciated their comfortable quarters. The wards on Thursday looked even more bright than usual, owing to a prize having been offered for competition between the nurses allotted to the different wards for the prettiest floral decoration. Mr. W. Paul, who acted as judge, awarded the first prize to the Chandler Ward, the second falling to the Porter Ward, and the prizes were presented by the Princess Louise to the nurses belonging to these wards. The inspection of the wards was followed by a short concert, in which Mr. and Mrs. Henschel, Mrs. Semon, Madame Norman-Neruda, and Miss Janotha took part, their performance being listened to with evident pleasure by a large company. Subsequently Her Royal Highness visited a flower show held in the grounds of the institution, consisting of a beautiful display of cut roses, grown at the nursery of Messrs. Paul and Son, Waltham, where she graciously allowed the name of "The Marchioness of Lorne" to be given to one of Mr. Paul's new seedlings, a large rose of a bright carmine colour with no shading, the colour being even throughout. In the main building there was also a splendid display of cut flowers from Messrs. T. Ware's nurseries. After the departure of the Princess the fête was continued in the evening, when the hospital was brilliantly illuminated, the effect of the fairy lamps scattered among the flowers being very striking. A dramatic entertainment entitled *Between Two Stools* was presented, and in addition to the many other attractions there were exhibitions of scientific instruments, electric-lighting apparatus, medical electricity, and of the interesting machine called the writing telegraph. The arrangements closed with an organ recital by Mr. Boyes in the chapel.

Richmond Hospital shows in a striking way what can be done by means of collecting boxes. In 1872 the boxes placed in various shops, offices, and inns, realised £5 9*s.* 7*d.* for the benefit of the hospital; last year they brought in £313 11*s.* 7*d.* The boxes are cleared in June and December, and all sums over two shillings are acknowledged. Many of the boxes produce but little over this, but in some the contributions are of very respectable amount. That in the Fire Engine House at Richmond contained, when opened at the end of last month, £14 1*s.* 6*d.*, contributed during the half-year. The collections for this year amount as yet to £139 12*s.* 9*d.* We hope the six months on which we have just entered will show as good a result.

The first annual report of the committee of management of the London Skin Hospital was laid before the subscribers on the 5th at the Hotel Métropole. The chair was occupied by the Marquis of Carmarthen, M.P. Letters of regret for inability to attend were read from Sir Richard Owen, K.C.B., Sir Alexander Armstrong, K.C.B., F.R.S., Dr. J. S. Bristowe, F.R.S., Mr. Ernest Baggallay, J.P., Mr. James J. Fellows (agent-general for New Brunswick), and others. Amongst those present were the Rev. Geo. Blake Concanon, D.D., Mr. H. Oakley, Mr. Marks, Mr. Chas. Pritchard, Mr. J. G. Albert, and several ladies. The hospital was established in March, 1887, for the treatment of skin diseases amongst the poorer classes, and as a school for instruction in the science and practice of dermatology. The institution is entirely free to the necessitous, and also affords relief to those above that class who are unable to pay the ordinary medical fees. During the past year there were admitted 725 out-patients, who made 3,398 attendances. Many applications have been received for admission as in-patients; but at present the funds at the disposal of the committee have not been sufficient to enable them to open an in-patients' department. The noble chairman made a strong appeal on behalf of the hospital, and said there are liabilities amounting to £369, while the cash in hand and at the bank only amounted to £51 7*s.* 6*d.* This is mainly due to the preliminary expenditure. It was mentioned that the hospital is almost entirely dependent on voluntary contributions.

THE following vacancies are announced this week, the amount of salary in each case being given after the name of the institution :—

**Resident Medical Officers.**

Central London Ophthalmic Hospital, Gray's Inn Road. House Surgeon.—Board; no salary.
Cumberland Infirmary, Carlisle. House Surgeon.—£70 per annum, and pupils' fee, with board, &c.
Cumberland Infirmary, Carlisle. Assistant House Surgeon.—£40 per annum, and pupils' fee, with board.
Glasgow Hospital for Sick Children. Assistant House Surgeon.
Hospital for Consumption and Diseases of the Chest, Brompton. House Physician.
North Staffordshire Infirmary. Assistant House Surgeon (for six months).—Board; no salary.
Queen's Hospital, Birmingham. Resident Physician.—£50 per annum, with board, &c.

**Non-resident Medical Officers.**

Belgrave Hospital for Children, 79, Gloucester Street, S.W. Surgeon to Out patients. Candidates must be F.R.C.S.
Bristol General Hospital. Assistant Surgeon. Candidates must be F. or M.R.C.S., or Graduate in Surgery of some University of the United Kingdom.
Chelsea Hospital for Women, Fulham Road. Three Clinical Assistants.—£5 5s for three months.
City of London Hospital for Diseases of the Chest, Victoria Park, E.—Assistant Physician. Candidates must be F. or M.R.C.P., Lond.
Killybegs and Tribane. Admiralty Surgeon and Agent for these Coastguards' Stations —£50 per annum.
Middlesex Hospital, W. Physician for Skin Diseases.
Parish of Edrachillis, Sutherland. Medical Officer.—£150 per annum, with free house.
Parish of Kirkmabreck, Kirkcudbrightshire. Medical Officer for the Poor.—£35 per annum.
Parish of St. George-in-the-East. Medical Officer for Infirmary and Workhouse.—£300 per annum, with unfurnished house.
Plymouth Public Dispensary. Physician's Assistant.—£60 per annum. Candidates must be doubly qualified.

---

THE Seaside Convalescent Home at Seaford, Newhaven, Sussex, officially established as a public meeting at the Mansion House in 1860, has for nearly thirty years been giving the benefits of perfect rest, pure air, liberal diet, and sea-bathing, when necessary, to poor convalescents of both sexes and all classes and creeds. More than 12,600 persons, comprising a large number of dwellers in the great metropolis—by occupation, clerks, artisans, policemen, postmen, apprentices, male and female servants, mothers of families, shop and machine hands—have been treated at Seaford for four weeks or for more, with such excellent results, that applications for the society's help far exceed its capacity. The demand for admission to the hospital in the summer months becomes so great that often a large proportion of poor patients are often unable, owing to the congested state of the hospital, to obtain admission for a month, and sometimes longer. In view of this sad state of things the committee are most anxious to add at least 30 more beds to the institution, for which it will be necessary to raise a sum of over £6,000 for building and sustentation. Many liberal friends have promised assistance, and the managing committee are now earnestly soliciting the co-operation of all public bodies, firms, and individuals in aiding them to increase the hospital, and thus overcome the difficulty under which they labour. This hospital has proved successful, even beyond the anticipation of the founders, in repairing the ravages of fevers and other serious maladies, and in assisting recovery after grave surgical operations and dangerous accidents. Many, indeed, not only recover from their sickness under the care of this charity, but attain a degree of health that the ordinary conditions of their lives had not permitted them to hope for, and it is to be hoped that lack of funds will not force the committee to suspend or lessen their beneficent work. Mr. Frank Maitland is the secretary to the Home, and the committee's offices are at 36, Southampton Street, Strand, where all information can be obtained.

THE distribution of prizes, medals, and certificates to the students of the medical school in connection with St. Thomas's Hospital, took place on Thursday afternoon, Professor Stokes, M.P., in the chair. The prizes were distributed, and among those who had specially distinguished themselves may be mentioned Mr. J. E. Harris, who won the the Entrance Science Scholarship (value 125 guineas) and certificate of honour; Mr. C. H. James, who received Solly medal and prize; Mr. F. C. Abbott, Mr. W. B. Winston, and Mr. H. G. Turney. Dr. Bristowe made a few remarks, in which he said the number of students now attending the school was nearly 400. Professor Stokes, M.P., then briefly addressed the students and the company present.

---

THE ceremony of cutting the first sod for the foundations of the new Victoria Infirmary, to be erected at Langside, took place on Wednesday afternoon in very unfavourable weather, at Glasgow. Rain began to fall heavily just as the proceedings in the open air commenced, and continued until the ceremony was finished. The site chosen for the new infirmary is on the sloping ground to the south-east of the Queen's Park, immediately adjoining the Langside Established Church, and is eminently suited, from its elevated position, to display the new buildings to advantage and secure a dry and bracing atmosphere. The scheme for the erection of an infirmary for the South Side of Glasgow was set on foot at a meeting of the inhabitants of the city and suburbs, held in the Merchants' Hall, Glasgow, on February 28th, 1887, which was largely attended, and was presided over by the Lord Provost. At that meeting Mr. Renny Watson stated that a committee had been appointed in 1881 to take steps for the erection of the infirmary, and up to that time, although no general canvass had been made, they had obtained subscriptions to the extent of over £8,000. The committee felt that they could not make a fair beginning on less than about £20,000. Resolutions were afterwards carried resolving to go on with the scheme, and to take immediate steps for raising the necessary funds. The subscriptions up to the present time amount to about £17,000, and in addition to that sum a present payment of £10,000 is to be made from the Couper Trust. The late Mr. Couper left the residue of his estate, amounting to £50,000, to the infirmary, but his widow was life-rented on it. She has, however, generously offered to hand over this £10,000 for the benefit of the institution at once. The portions of the infirmary which the committee intend to erect at present, comprise the administrative department fronting Langside Road, and the pavilion block immediately behind, which will contain ample accommodation for sixty patients. The administrative department will be sufficient for the infirmary when completed, but it is intended to utilise the rooms which will not meantime be required by the infirmary officials as private wards for paying patients. While these buildings will be complete in themselves, the plan of the infirmary, consisting as it does of a series of pavilions, lends itself naturally to extension from time to time by the addition of one pavilion after another as may be necessary. The party gathered on a wooden platform erected on the ground for the occasion, amongst those present being: Mr. W. Renny Watson, chairman of the Executive Committee; ex-Lord Provost M'Onie, Provost Marshall, Provost Hunter; Bailies Morrison and Mayberry, ex-Bailie Laing; Councillors Paterson and Walter Wilson; Deacon-Convener Tullis; Collector Mason; the Rev. Dr. Smith, Cathcart; the Revs. Mr. Ritchie, Mr. Niven, and Mr. Muir; Drs. Kelly, Napier, Pollok, and Scott; Captain M'Laren, and many others. Apologies for absence were intimated from Sir James King, Lord Provost; ex-Lord Provost Ure; Mr. J. Campbell White; Mr. J. Gordon, of Aitkenhead; Mr. T. R. J. Logan, the City Chamberlain, Dr. J. B. Russell, Dr. Duncan, Provost Browne, and a number of the magistrates and members of the Town Council.

